# Arctic cryosphere and Milankovitch forcing of Great Basin paleoclimate

**DOI:** 10.1038/s41598-017-13279-2

**Published:** 2017-10-11

**Authors:** Matthew Lachniet, Yemane Asmerom, Victor Polyak, Rhawn Denniston

**Affiliations:** 1 Department of Geoscience, University of Nevada Las Vegas, 4505 S. Maryland Pkwy, Las Vegas, NV 89154 USA; 2 Department of Earth and Planetary Science, University of New Mexico, 221 Yale Blvd. NE, Albuquerque, NM 87131 USA; 3 Department of Geology, Cornell College, 600 First Street West, Mount Vernon, Iowa 52314 USA

## Abstract

Although Great Basin paleoclimate history has been examined for more than a century, the orbital-scale paleoclimate forcings remain poorly understood. Here we show – by a detailed phasing analysis of a well-dated stalagmite δ^18^O time series – that Great Basin paleoclimate is linearly related to, but lagged, the 23,000 yr precession cycle in northern hemisphere summer insolation by an average of 3240 years (−900 to 6600 yr range) over the last two glacial cycles. We interpret these lags as indicating that Great Basin climate is sensitive to and indirectly forced by changes in the cryosphere, as evidenced by fast and strong linkages to global ice volume and Arctic paleoclimate indicators. Mid-latitude atmospheric circulation was likely impacted by a northward shifted storm track and higher pressure over the region arising from decreased sea ice and snow cover. Because anthropogenic warming is expected to reduce northern hemisphere snow and ice cover, continued increase in atmospheric greenhouse gases is likely to result in warming and drying over coming centuries that will amplify a warming trend that began ~2400 years ago.

## Introduction

**Figure 1:**
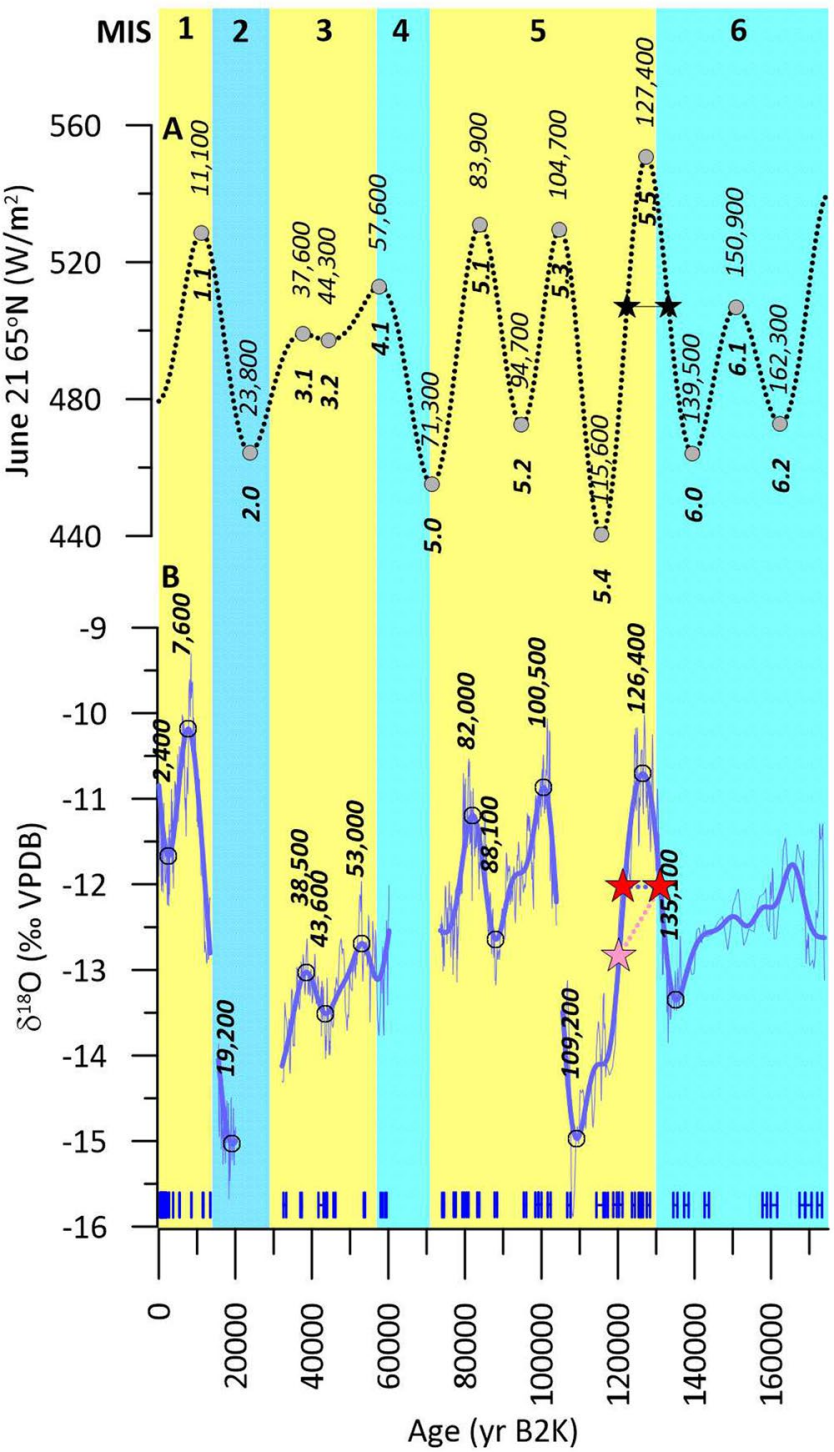
Phasing relationships for insolation and Great Basin Paleoclimate. (**A**) Is 65°N June 21 insolation; (**B**) is Leviathan chronology and band-pass filter; U-series ages are shown in blue for Leviathan chronology. Yellow and blue backgrounds are marine isotope stages 1–6. Circles are ages of peaks and troughs in band-pass filtered data. Stars indicate TII and inceptions (see text).

The orbital theory of climate change first elaborated in the 19^th^ century^1^ was subsequently confirmed to exert a strong control on global climate, as evident from pioneering paleoceanographic studies showing regular orbital obliquity (41,000 yr cycles) and precession (19,000–23,000 yr cycles) in benthic foraminifera δ^18^O associated with changing ice volume on the continents^2,3^. The orbital paradigm suggests that Northern Hemisphere Summer Insolation on June 21 at 65°N latitude (hereafter NHSI) controls global climate via variations in ice sheet extent, which in turn exert responses in temperature and atmospheric and oceanic circulation. However, in many continental regions the role of orbital variations in paleoclimate remains poorly known due to the difficulty of obtaining long absolutely dated paleoclimate records. For example, controls on Great Basin paleoclimate have been debated since the classic 19^th^ century monographs on Pluvial Lakes Lahontan and Bonneville^4–7^ (Fig. 1). Despite this work, a lack of highly resolved records spanning orbital timescales makes it difficult to attribute the past controls on Western North America (WNA) paleoclimate and to facilitate future projections.

An exception is the well-dated 175,000 yr-long^8^ speleothem oxygen isotope record from caves in Nevada, based on records from Leviathan, Pinnacle, and Lehman Caves^8,9^. Several features of the Leviathan chronology suggest that it is a reliable proxy for Great Basin climate variations: it is securely dated with 65 high-precision U-series ages; the stalagmites grew in near-surface vadose zone caves <100 m from the land surface in areas of rapid infiltration of winter precipitation, ensuring a short response time to atmospheric climate changes, the predominant source of aquifer recharge; and modern stalagmite calcite forms in isotopic equilibrium with cave drip waters^8^. Further, the δ^18^O time series showed a strong NHSI control on continental paleoclimate, with climate lagging insolation by a few thousand years. However, the origin and quantification of this lag has not been completed, hampering our ability to identify plausible forcing mechanisms. Here, we investigate the orbital-scale phasing relationships between Nevada paleoclimate and insolation forcing over the past 175,000 years, and highlight several key features of the δ^18^O time series that support a fast climate response to precessional-scale forcing in the Great Basin of western North America.

## Milankovitch phasing relationships

**Figure 2:**
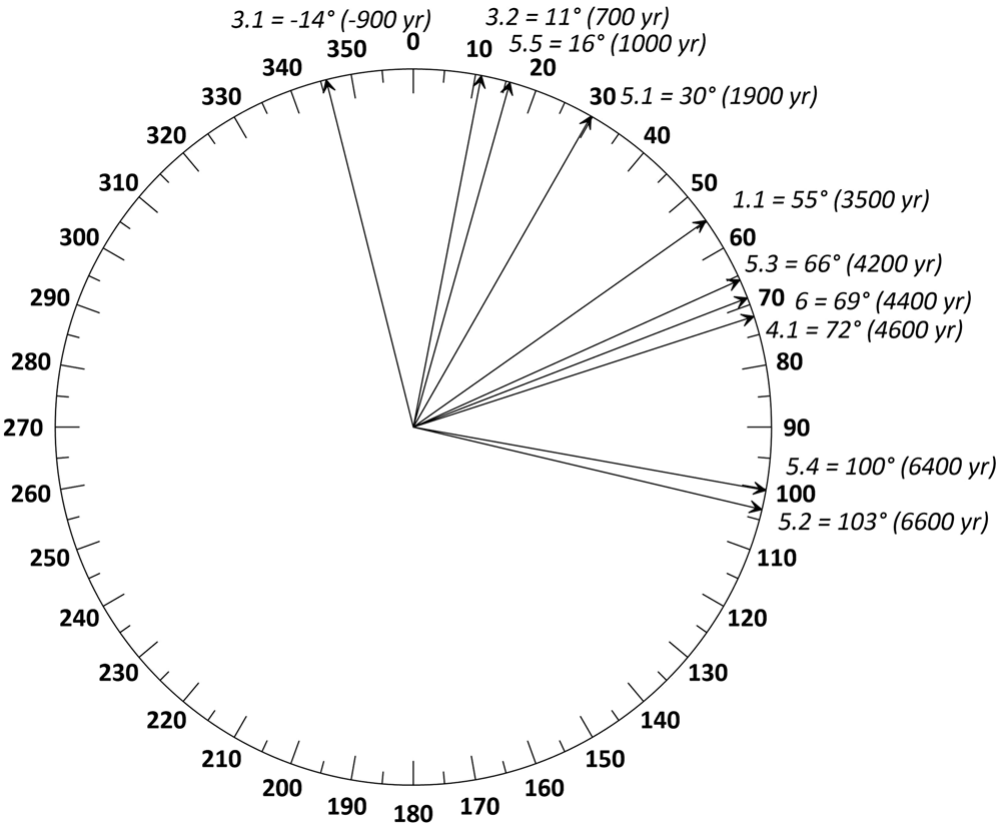
Great Basin paleoclimate lags insolation forcing. Phase wheel diagram (in ° relative to 23,000 yr precession cycle) showing phasing differences between June 21 insolation at 65°N and δ^18^O_ivc_ of the Leviathan chronology, positive phasing means δ^18^O_ivc_ lags NHSI. Labels are insolation substages and lags in years of Leviathan behind insolation forcing. See Table 1.

Following ref.^10^, we use the classic Milankovitch forcing of the June 21 65°N insolation curve^11^ to quantify phasing relationships between insolation and WNA paleoclimate as registered in the low-pass filtered Leviathan chronology ice volume-corrected record (δ^18^O_ivc_, see methods). We use insolation sub-stage (ISS) nomenclature, which labels peaks and troughs in NHSI analogously to extrema in Marine Isotope Stage (MIS) numbering wherein, for example, the last glacial maximum is ISS 2.0 and the penultimate interglacial is ISS 5.5 (Fig. 2 and Table 1). At our study sites in the Great Basin, lowest meteoric precipitation δ^18^O values are associated with cold winter storms delivering high-latitude moisture, and warmer low-latitude moisture sources have high δ^18^O values^8^. Precipitation amount does not affect δ^18^O values in modern precipitation events^8^. These precipitation δ^18^O signatures are then recorded in stalagmite calcite. Summer precipitation is a minor (<10%) component of cave infiltration^12^, and so we interpret the speleothem δ^18^O_ivc_ variations in the past as a proxy for wintertime temperature and moisture source variations, and our vadose-zone cave locations are unaffected by changes in groundwater levels.

**Table 1:** Timing of insolation substages and correlative climate variations in Great Basin speleothems.

Insolation substage	Age yr B2k (ISS)	Jun 21 inso (w/m^2^)	Age yr B2k (Leviathan)^1^	Leviathan δ^18^O_ivc (‰ VPDB)_	Δage^2^ (ISS - Leviathan; yr)	Age uncertainty^4^ (2σ, yrs)
1.0	—	—	2400	−11.7	—	20
1.1	11,100	529	7600	−10.2	3500	50
2.0	23,800	464	(19,200)^3^	−15.0	(4600)^3^	430
3.1	37,600	499	38,500	−13.0	−900	470
3.2	44,300	497	43,600	−13.5	700	300
4.1	57,600	513	53,000	−12.7	4600	320
5.1	83,900	531	82,000	−11.2	1900	460
5.2	94,700	472	88,100	−12.6	6600	670
5.3	104,700	530	100,500	−10.9	4200	610
5.4	115,600	440	109,200	−15.0	6400	920
5.5	127,400	551	126,400	−10.7	1000	650
6.0	139,500	464	135,100	−13.4	4,400	890

^1^Ages are determined on peaks and troughs of the precessional-scale 23,000 yr zero-phase band-pass filters on a 100-yr timestep. ^2^For Δage, positive values mean a lag; ^3^Age of ISS 2.0 feature in Leviathan chronology is uncertain because of hiatuses bracketing this time interval; age estimate is for comparison only. ^4^Age uncertainties were interpolated from age model uncertainty of the individual stalagmites and rounded to nearest 10 yr.

δ^18^O_ivc_ values for the Leviathan chronology show pronounced orbital-scale variability that exhibit clear correlative peaks to NHSI (Fig. 1), with the strongest fits to the June 21, July 21, and August 21 curves (Figure S1), each of which matches the unique triplet of low/high/very low insolation during ISS 6.0/5.5/5.4. We show for the first time the phasing relationships between climate and NHSI over the last two glacial cycles. Out of ten insolation-correlative features in the δ^18^O_ivc_ record, nine show delays (from 700 to 6600 yr) to NHSI, and the lags exceed the 2σ age uncertainties determined from time series age model uncertainties (Table 1; see methods). Only ISS 3.1 appears to lead NHSI (by ~900 yr). Considered together, the average phase lag for the 175 ka Leviathan record is 3240 years with a range of −900 to 6600 years, approximately 25% smaller than the 4800 years by which global ice volume lags precession^13^. The shorter lag time for Leviathan than global ice volume may be related to chronological uncertainties with the marine benthic δ^18^O record, but it may also reflect the faster response time of Great Basin paleoclimate to sea ice and snow cover changes in the Arctic, which would have responded more rapidly to insolation changes than ice sheet extent. We also show for the first time that Great Basin δ^18^O_ivc_ is linearly related to NHSI, suggesting a linear response (r = 0.89, p-value = 0.002) between forcing and paleoclimate in the Great Basin (Figure S2).

The filtered Great Basin δ^18^O_ivc_ time series is particularly important because it informs understanding of past glacial intervals and the projected length of the current interglacial^14^ and future climate changes. Our data closely constrain the maximum cooling during the penultimate glacial maximum (PGM) to 135,100 yr B2k (δ^18^O_ivc_ = −13.35‰), lagging NHSI by 4400 years (Figure S3), and could be considered a type-age for correlation of other paleoclimate records in WNA with lower-precision dating^15^ such as cosmogenic nuclide dated glacial events. Peak ISS 5.5 δ^18^O_ivc_ (−10.71‰) at 126,400 yr was delayed 1000 years behind insolation. We also show that the onset of the last interglacial lagged insolation: Termination II (TII), as defined by the mid-point δ^18^O_ivc_ value of −12.03‰, dates to 131,100 yr B2k (Fig. 1 and Table 1) but dates to 133,200 in NHSI. The last glacial inception began at 121,300 yr B2k, for an interglacial optimum (equivalent to MIS 5e) duration (*sensu stricto*) of 9800 years using the TII δ^18^O_ivc_ threshold (Table S1). We also estimate the last glacial inception age based on the δ^18^O_ivc_ mid-point for the ISS 5.5/5.4 extrema (−12.84‰ at 120,300 yr B2k), providing a last interglacial duration (*sensu lato*) of 10,800 years. These durations are close to those predicted for Milankovitch forcing, being approximately ½ the 19,000 and 23,000 year precession cycles, respectively. The timing of glacial and stadial periods also fits well with precessional-scale orbital forcing. We lack continuous data for the Last Glacial Maximum and are thus unable to precisely estimate phase relationships, but a likely timing based on the filtered δ^18^O_ivc_ of 19,200 yr B2K suggests a 4300 year lag behind NHSI (Fig. 1). More striking is the 6400-year lag during ISS 5.4 (correlative to MIS 5d), when very low δ^18^O_ivc_ values indicate cold conditions in the Great Basin following an extreme insolation minimum. For the Holocene (ISS 1.1), filtered Leviathan δ^18^O_ivc_ peaked at 7600 yr B2K, 3500 years after NHSI at 11,100 yr B2K. Unfiltered δ^18^O_ivc_ peaks earlier (8380 yr B2K), giving a minimum lag of ~2700 years. In an unusual departure compared to past behavior where δ^18^O_ivc_ tracked insolation closely, late Holocene δ^18^O_ivc_ has been increasing since ~2400 yr B2K while insolation has decreased.

## Global and regional paleoclimate connections

If insolation were directly forcing Great Basin paleoclimate via local temperature, we would expect to see nearly synchronous speleothem climate variations in response to changing NHSI. The millennial-scale δ^18^O_ivc_ lag behind NHSI suggests that the Great Basin climate response is instead indirect and mediated through one or more fast-response (relative to precessional-scale insolation forcing) components of the climate system. We investigate several pathways by which insolation forcing is linked to Great Basin climate: global ice volume^13^, northern hemisphere temperature^16^, regional sea surface temperature (SST) in the California Current system off WNA^17^, Arctic sea-ice variations^18^, radiative forcing from greenhouse gases^19,20^, and high latitude temperatures from Greenland^21^ and Antarctic ice core data^22^.

**Figure 3:**
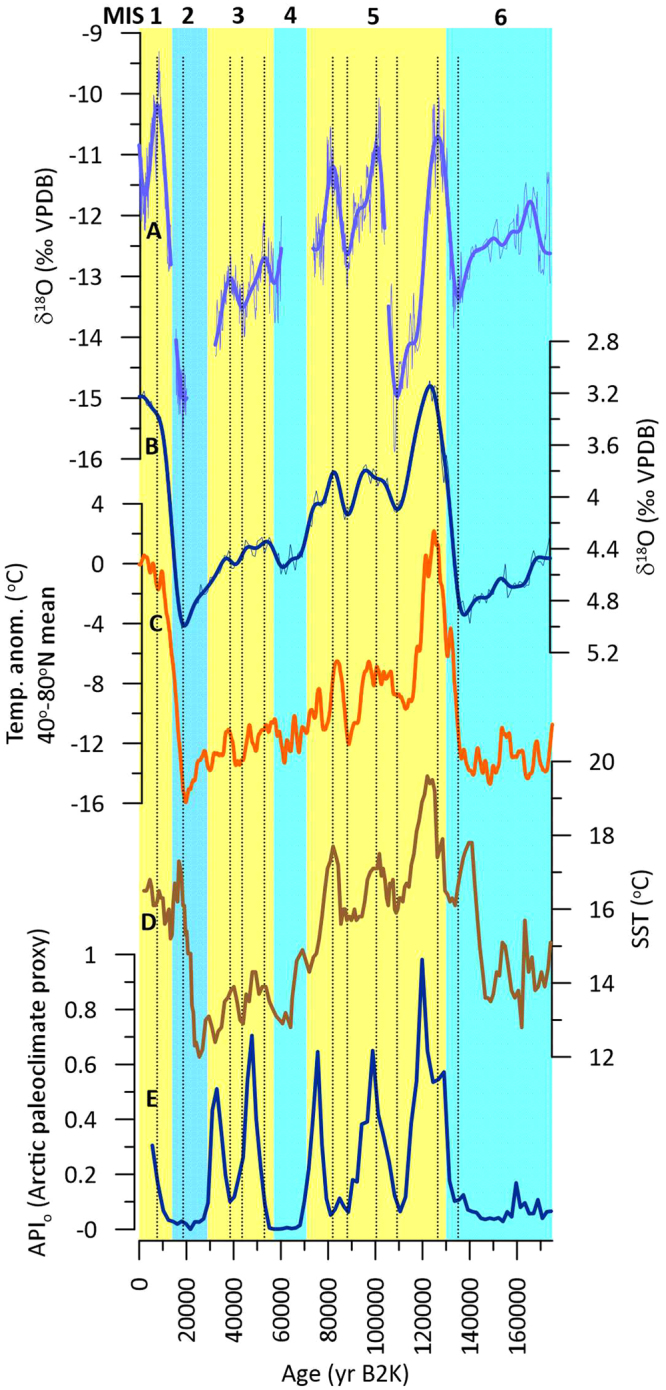
Great Basin paleoclimate is linked to the state of the cryosphere. (**A**) Leviathan chronology; (**B**) global ice volume (LR04 stack)^13^, (**C**) northern hemisphere temperature^16^, (**D**) California Current SST from site ODP 1012^17^, and (**E**) Arctic paleoclimate index^18^. Vertical dotted lines are ages of Leviathan δ^18^O_ivc_ maxima and minima for comparison to other records. Yellow and blue backgrounds are marine isotope stages 1–6.

The Leviathan chronology shares strong similarities with the global benthic foraminifera δ^18^O stack^13^, a proxy for global ice volume, salinity, and deep water temperature (Fig. 3). Much of the saw-tooth structure of Great Basin paleoclimate variations may thus have arisen from temperature and atmospheric circulation changes associated with the growth and decay of the northern hemisphere ice sheets, which are expected to exert a particularly strong influence on regional temperature and atmospheric circulation^23^. Both Leviathan and global ice volume show cooling trends between 175,000 and ~137,000 yr B2K as ice sheets expanded, with subsequent minimum interglacial ice volume lagging Leviathan by 3300 years. The onset of the last interglacial and duration of ISS 5.5 (MIS 5e) also followed NHSI, demonstrating that precessional-scale insolation forced deglaciation in WNA over TII. The peak cold conditions during the ISS 5.4 δ^18^O_ivc_ minimum align closely with increased global ice volume (a benthic δ^18^O maximum). We also observe a strong similarity between the Leviathan chronology and an estimate of northern hemisphere temperature (40–80°N) derived from an ice sheet model and the LR04 stack^16^. In contrast, we observe several major discrepancies with California Current alkenone SST (core ODP-1012). We conclude that the Leviathan chronology’s δ^18^O_ivc_ variations are strongly linked to global climate evolution on glacial/interglacial timescales, but are disconnected from regional ocean temperatures off WNA, at least on the orbital scale.

**Figure 4:**
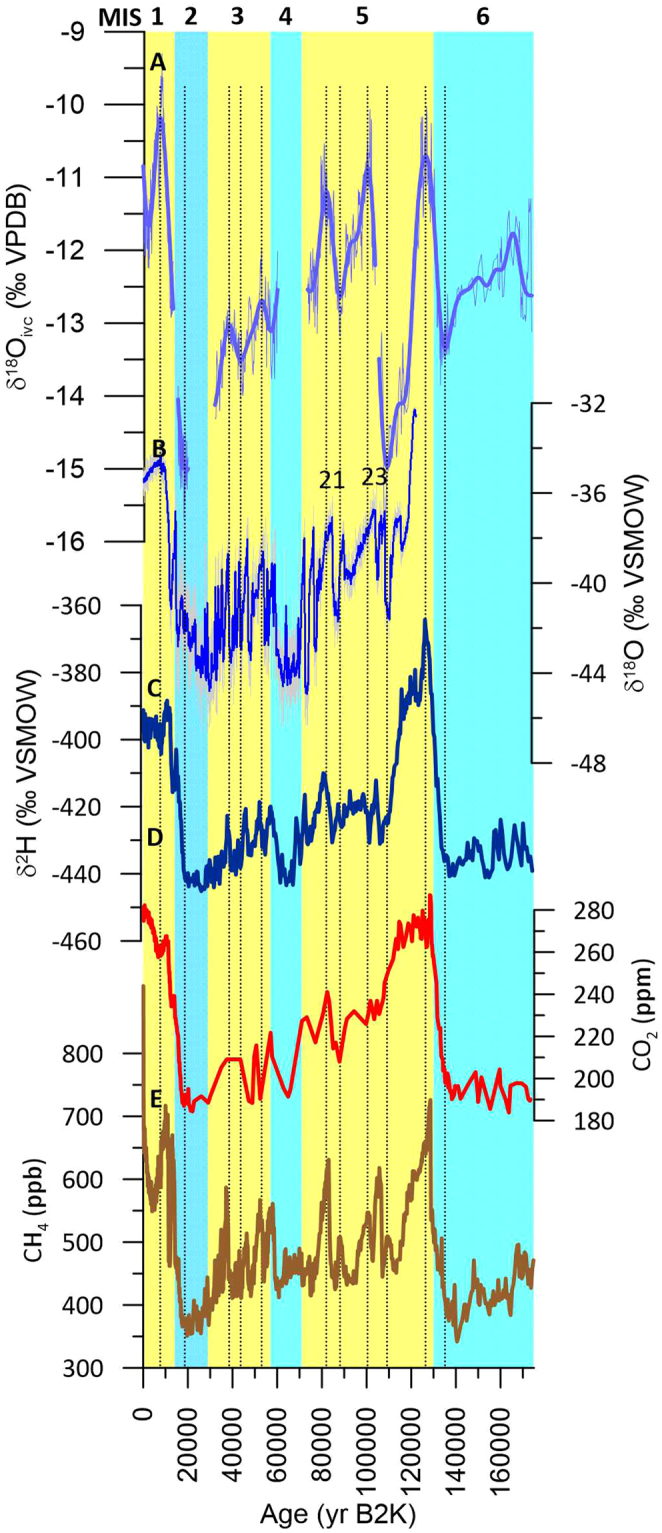
Comparison of Leviathan chronology to ice core stable isotope and greenhouse gas records from Greenland and Antarctica. (**A**) is Leviathan chronology, (**B**) is NGRIP^21^ δ^18^O (with Greenland interstadials 21 and 23 indicated), (**C**) is EPICA Dome C Antarctica δ^2^H (ref.^22^), (**D** and **E**) are Dome C CO_2_ and CH_4_ concentrations^19,20^, respectively. Vertical dotted lines are ages of Leviathan δ^18^O_ivc_ maxima and minima for comparison to other records. Yellow and blue backgrounds are marine isotope stages 1–6.

Links to Arctic paleoclimate are evident between the Great Basin and the NGRIP δ^18^O ice core record (GICC05 extended chronology)^21^. Peak cold conditions in ISS 5.4 were synchronous in both locations, and both records show a small warming ‘shoulder’ at ~114,600 yr B2k (Fig. 4). The transition from ISS 5.3 into 5.2 following Greenland interstadial (hereafter GIS) 23 shows a similar gradual cooling trend, compared to the more rapid rate of cooling associated with the ISS 5.1 to 5.0 transition following GIS 21. Similarity of MIS 5 δ^18^O time series between Greenland and the Great Basin suggest commonalities of paleoclimatic change when global ice volume was low. In contrast, the Leviathan chronology does not preserve evidence of a one-to-one pairing of NGRIP δ^18^O millennial-scale events in MIS 3 when ice volumes were intermediate, as apparently manifested in lake^24^ and speleothem^25^ records. A possible explanation of this observation is that such events contained summer influences associated with changing seasonality, whereas our cave sites appear to be almost exclusively winter-sensitive.

## Northern Hemisphere cryosphere forcing of Great Basin paleoclimate

Although our phasing analysis indicates a high sensitivity of the Great Basin to high-latitude ice sheet forcing, two prominent periods are not easily explained by this relationship: the pronounced δ^18^O_ivc_ minimum at ISS 5.4 (MIS 5d), and decreasing Holocene δ^18^O_ivc_. How could climate be so apparently cold in the Great Basin in ISS 5.4 when global ice volume was small and northern hemisphere temperatures were warm? Rising greenhouse gas concentrations and reduced North American ice sheet extent (Fig. 3) would be expected to lead to warm ISS 5.4 temperatures, in contrast to low and cold δ^18^O_ivc_ values. To test the idea that the forcing may reside in the fast-response variation of northern hemisphere sea ice, we compared the Leviathan δ^18^O_ivc_ to the Arctic Paleoclimate Index (API_o_), which characterizes ostracod density in Arctic ocean sediments^18^, which in turn were forced by sea-ice mediated productivity changes. The API_o_ shows high productivity during high NHSI, and vice versa for low NHSI. A major productivity decrease during ISS 5.4 suggests a glacial-like sea-ice expansion despite intermediate global temperatures and ice volume, and is supported by cold climate conditions in the Siberian Arctic^26^ at this time when permafrost ice wedge growth was vigorous. A decrease in high latitude temperature^27^ and expanded snow fields^2,28^ coincident with the ISS 5.4 insolation minimum may have driven sea ice expansion and amplified Arctic cooling, and both cryosphere indicators exhibit a fast response to orbital forcing. The low ISS 5.4 δ^18^O_ivc_ in Leviathan could be interpreted as forced by an increase in Arctic sea ice extent and high-latitude snow fields driven by the period’s lowest temperatures and summer insolation. Such a contention is supported by the strong influence between NHSI and Arctic summer sea ice extent (and weak influence from greenhouse gas forcing) in modeling studies^29^. Stadial conditions during ISS 4.0 and 2.0 were associated with a cessation of growth in stalagmite LC-1 from possible periglacial conditions overlying the high-altitude (2400 m) Leviathan Cave^8^, and both hiatuses also coincided with pronounced Arctic productivity minima and high sea-ice extent (Fig. 3).

**Figure 5:**
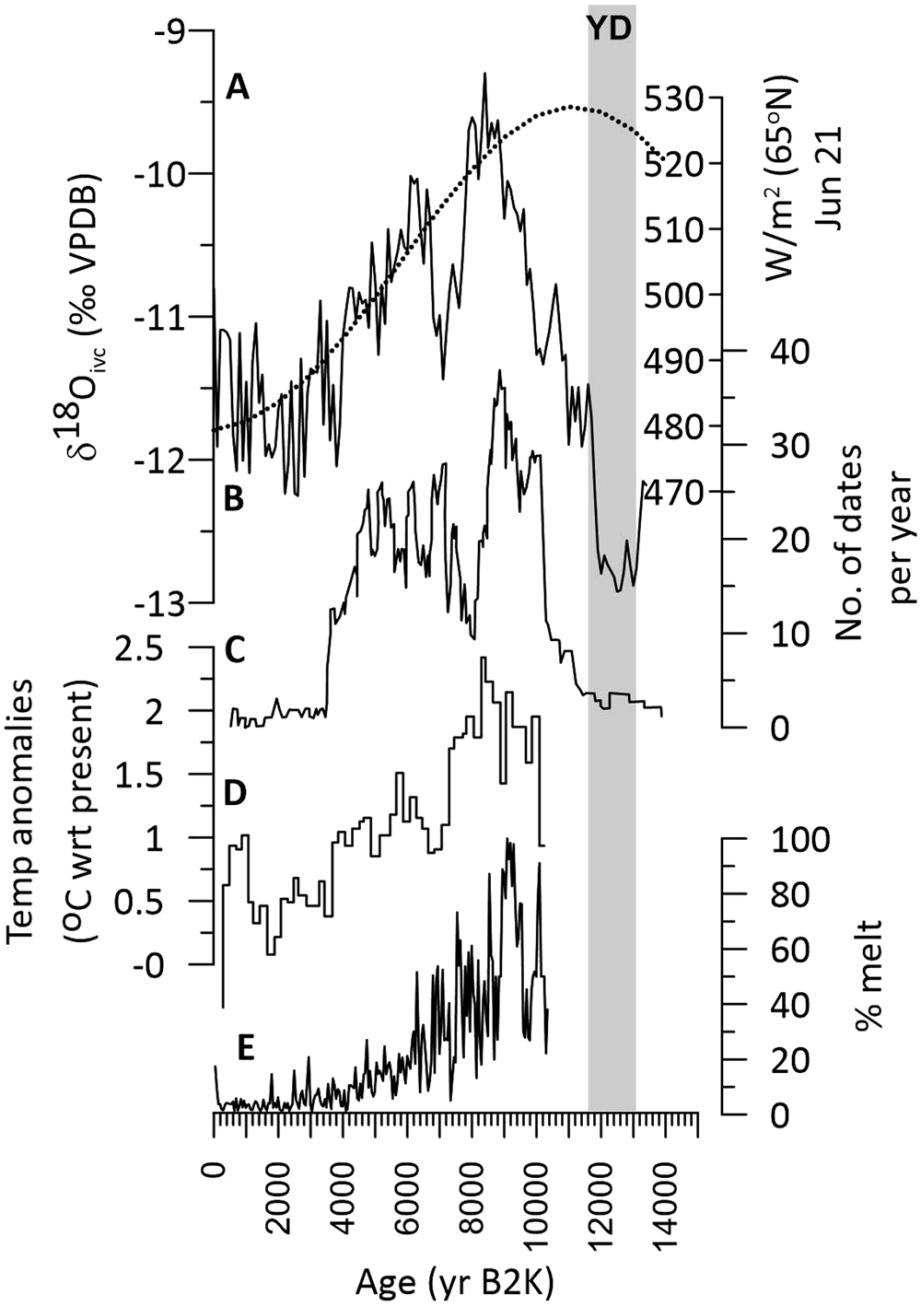
Great Basin links to the Arctic over the Holocene. Comparison of (**A**) unfiltered Leviathan δ^18^O_ivc_ and (**B**) insolation, (**C**) fossil tree radiocarbon dates at the Russian treeline^32^, (**D**) pollen-based summer temperature anomalies in the Western Canadian Arctic^31^, (**E**) and melt layer percentage in the Agassiz ice cap^30^ shows an orbital-scale control on Holocene Arctic and Great Basin paleotemperature. YD indicates the Younger Dryas chronozone.

We also observe a prominent (2.9‰) decrease in δ^18^O_ivc_ from ca. 8400 ka to 2400 yr B2k, indicative of increasingly colder winters, despite the lack of North American ice sheet growth. Likewise, stable or rising northern hemisphere temperatures and increasing carbon dioxide and methane concentrations (Fig. 4) cannot easily explain cooling in the Great Basin. Because NHSI decreased in tandem with δ^18^O_ivc_, we suspect that cryosphere changes in the Arctic may be responsible. Two indicators of Arctic warmth are the presence of melt layers in the Agassiz ice cap on Ellesmere Island, Canada^30^, which were most abundant in the early Holocene (ca. 9.1 ka) and pollen evidence for decreasing Holocene summer temperatures in the Western Canadian Arctic^31^ (Fig. 5). An increase in fossil trees near the Russian Arctic treeline^32^ also indicate early Holocene warmth and subsequent decline that synchronously tracks Leviathan cooling. All records transition to cooler conditions during the late Holocene coincident with falling NHSI. The Holocene trends are substantiated by proxy evidence for an extended Arctic sea ice minimum from ca. 8550 and 6000 years ago^33^, widespread evidence for late Holocene Arctic cooling^34–36^ and sea-ice expansion^37^, and model results^38,39^ demonstrating an insolation forcing of sea-ice extent^29,40^.

Because Great Basin climates lagged insolation even during periods of low global ice volume (e.g., during ISS 5.5–5.1), it is difficult to attribute forcing solely to ice sheet effects on northern hemisphere paleoclimate. Changes in both high latitude sea ice and snow cover are likely to influence the mid latitudes via atmospheric teleconnections^41^. Modern observations suggest that the impact of increasing snow cover may have resulted in decreased atmospheric pressure and increases in storm track intensity and cyclogenesis in the eastern North Pacific Ocean^42^. Such forcings would result in stronger storms that deliver greater winter precipitation to the Great Basin. In contrast, a reduction in modelled sea ice extent resulted in pronounced winter precipitation deficits, by up to 50%, in the Great Basin and Western U.S.^41^. The reduced sea ice extent increased the 500-mb geopotential height, diverting storms away from the southwest to higher latitudes, resulting in a northward shift of the mid latitude storm track^43^. During winters following sea ice lows from the previous summer, the atmosphere may ‘remember’ the summer sea ice extent because of a lowered mid- to high-latitude temperature gradient, which weakens the Aleutian Low^44^ and results in fewer and less intense storms reaching the Great Basin. A strengthening of the subtropical highs arising from decreased sea ice and snow cover extents^43^ also results in fewer storms impacting the southwestern United States. This atmospheric configuration typically produces high pressure over the Great Basin and inhibits delivery of Pacific moisture to the Great Basin^45^ today, and we suggest that similar climate dynamics applied in the past. Similarly, increased snow cover extent results in cooling via the snow-albedo feedback^28,46^, and advection of cold high-latitude moisture to the Great Basin during periods of greater sea-ice and snow-field extent is a plausible explanation for the ISS 5.4 and Holocene cooling in the absence of significant ice sheet extent.

On long orbital timescales, similar relationships may also be operative: changes in atmospheric circulation as a result of insolation changes are largely mediated through sea ice and positive feedbacks in the Arctic^27^ driven by changes in the meridional temperature gradient. Our data strongly support the hypothesis that, on orbital time scales, reduction in sea-ice extent is associated with higher temperature and less precipitation over the Great Basin. Arctic sea ice extent likely exerts control on mid-latitude atmospheric circulation via a weakening of the jet stream associated with a decreased latitudinal temperature gradient.

The large and variable phase lag (−900 to 6600 yr) appears to be a robust feature of our data, as the lags are considerably larger than the age model uncertainties (Table 1). A possible explanation is that other seasonal insolation variations (Figure S1) may exert a control on Great Basin paleoclimate. For example, during ISS 3.1 (when high Leviathan δ^18^O_ivc_ at 38,500 yr B2k leads June 21 insolation by 900 years), it lags peaks in April 21 and May 21 insolation which arrive at 39,000 to 40,000 yr B2k, respectively. July 21 and August 21 insolation would reduce the lags at other time intervals. Clearly, reducing the complex Earth climate system to a single month of insolation is an oversimplification, a fact noted by previous workers^10^. In addition to variable seasonal sensitivity, the variable phase lag may also arise from multiple feedback processes in the climate system, each of which may have different time-variable response times to insolation forcing that depends on changing boundary conditions, e.g., ice-sheet size, the carbon cycle, Arctic Ocean temperatures, and the state of the biosphere. To test for this possibility, we find a weak but apparent negative correlation (r^2^ = 0.3 for a linear and 0.53 for second order polynomial fits) between Leviathan δ^18^O_ivc_ lags and the correlative NHSI value (Figure S5): the lags appear to be largest during glacial stages of ISS 2.0 (the LGM) and 6.0 (the PGM), and interglacial stages ISS 5.2 and 5.4, suggesting that the lags may arise in part from ice sheet influences on atmospheric circulation. We do not see similar correlations to CH_4_, CO_2_, or benthic δ^18^O, suggesting that insolation – likely mediated through sea ice and snow cover – is the driver of Great Basin paleoclimate. We also do not see any systematic influence of Great Basin background hydrologic state (e.g., pluvials and interpluvials) in the δ^18^O_ivc_ lag times. We would expect to see shorter lag times during pluvial climates if effective moisture variations in the Great Basin were driving faster infiltration rates into the cave systems, or if the δ^18^O_ivc_ values were influenced by local pluvial lakes. That we do not see such effects is likely due to the rapid response time of drips into the shallow vadose zone caves and a lack of any significant local control on precipitation δ^18^O values. Lag times in phreatic zone caves, such as Devils Hole, however, may have been influenced by such local hydroclimatic variations.

Our observations also have implications for broader Great Basin paleoclimate and the interpretation of the phreatic zone Devils Hole calcite record^47^. First, maximum expansion of mountain glaciers during the LGM was reached around 20,000 yr BP^48^, some four thousand years after minimum NHSI at 24,000 yr BP. Second, the formation of the iconic Great Basin pluvial lakes typically reached highest levels around 17,000 to 18,000 yr B2k associated with Heinrich stadial 1^49,50^, and which also lagged insolation. Both observations suggest that lake and glacier hydroclimatic indicators lagged insolation to similar degrees as Leviathan δ^18^O_ivc_ record, but the lake and glacier records were also associated with abrupt millennial-scale forcing associated with Heinrich stadials and the Bolling/Allerod interstadial^48,49^.

Finally, our vadose-zone cave climate record constrains atmospheric circulation changes that are later recorded in phreatic zone calcite at Devils Hole. The amplitude of the ice-volume corrected δ^18^O_ivc_ record (see Methods) in Devils Hole is significantly smaller (Figure S5) than Leviathan, suggesting that dispersion of the δ^18^O signal in aquifer water reduced the amplitude of climatically driven δ^18^O values. Such dispersion and delay of waters through the regional aquifer may also explain the lag of correlative interglacial δ^18^O_ivc_ peaks behind Leviathan. For example, peak ISS 5.5 δ^18^O_ivc_ at Devils Hole arrived 2400 years after Leviathan, (and 3400 years after peak insolation), and the Holocene δ^18^O_ivc_ peaks at ca. 5000 yr B2K, compared to 7600 yr B2K in the filtered Leviathan δ^18^O_ivc_ and 11,100 yr B2K for insolation. Further support for dispersion of the δ^18^O signal in the aquifer is that the last interglacial optimum (MIS 5e) duration is half again as long (17,500 yr) in Devils Hole as in surface climate. The timing of lags in Devils Hole may also be related to changes in hydroclimate via effective recharge rates in the phreatic aquifer, a possibility that merits further investigation. We conclude that Devils Hole δ^18^O represents a delayed and dampened response to surface climate variations; ages and durations of isotopic anomalies are thus minima and maxima, respectively.

## Future climate in the Great Basin linked to the Arctic cryosphere

Our conclusion of high sensitivity of Great Basin paleoclimate to the state of the Arctic cryosphere has important implications for future climate projections. The modern climate is characterized by rapidly rising greenhouse gas concentrations, and the Arctic is expected to rapidly transition to sea-ice-free summers^51^ in the 21^st^ century as it likely did during the mid-Holocene^52^. Such a sea ice reduction will perturb wintertime atmospheric circulation in the Great Basin, leading to dynamic warming and drying of winters similar to that observed in the instrumental record^45^. Additionally, human-caused warming will amplify the warming trend that began 2400 years ago, with mean annual temperature increases at our study area of 3–4 °C by 2080 under a moderate (RCP 4.5) warming scenario^53^. Model results indicate Great Basin winter precipitation decreases of up to 50% as a result of a northward migration of the winter jet stream^45^. Considering that the peak warming interval in the Great Basin of the early to mid-Holocene ‘altithermal’^4,54^ was one of intensely hot and arid conditions and the region was only able to sustain sparse human populations, the future of Nevada climate looks to be one of increasing warmth and drought. Without aggressive action to decrease the rate of greenhouse gas concentrations in the atmosphere, the future of the Great Basin is likely to be one of heating and drying.

## Methods

Ages on Nevada stalagmites were determined on a ThermoElectron Neptune multi-collector inductively coupled plasma mass spectrometer at the University of New Mexico Radiogenic Isotope Laboratory, and stable isotope values were determined on a Kiel IV automated carbonate preparation device at the Las Vegas Isotope Science laboratory at UNLV directly coupled to a ThermoElectron Delta V stable isotope ratio mass spectrometer in dual inlet mode^8^. The time series of the vadose zone stalagmite δ^18^O values were adjusted for the change in δ^18^O of the ocean (denoted δ^18^O_ivc_) using the reconstruction of^55^, and for differences in the isotopic composition of precipitation falling at Lehman and Pinnacle Caves (+0.36 and −1.73‰ VPDB, respectively) relative to Leviathan Cave following^8^. This adjustment results in a small change in δ^18^O values that does not affect the shape or timing of the composite isotope curve. The Leviathan chronology δ^18^O_ivc_ record presented here includes a correction for the age of nine stable isotope samples (and the derivative interpolated time series) beneath a hiatus at 290.5 mm. This changes the age of the last isotope sample (291 mm) before the hiatus to 73,647 from 69,622 yr B2k. The age correction does not change any interpretations of this or previous work. We estimated age uncertainties for individual stalagmite age models using the COPRA software^56^, and interpolated age uncertainties at the 95‰ level for each climate event in Table 1 in the Leviathan chronology.

Because the DH2-D record is significantly dampened relative to the Leviathan signal due to aquifer mixing and dispersion (Figure S4; see text for explanation), we corrected the measured δ^18^O values by changes in δ^18^O of the ocean that were scaled by a ratio of 0.4551, which is the ratio of the standard deviations of the Leviathan and DH2-D records, respectively. This ice volume correction more accurately reflects how a given precipitation isotopic signal is filtered and dampened via transit through the aquifer feedings Devils Hole better than the undampened correction^47^. Because of our different ice volume correction, the timings and amplitudes of DH2-D δ^18^O_ivc_ anomalies are slightly different than previously reported.

We then fit low-pass precessional-scale^2^ Butterworth filters to both δ^18^O_ivc_ records (sixth order with period of 23,000 years) with zero phase lag using the filtfilt function in Matlab. The low-pass filters were then used to constrain timings of isotopic events based on a peak and trough detection algorithm matched by correlation to June 21^st^ 65°N insolation for various insolation substages (numbered, 3.1, 3.2, etc.; analogous to Marine Isotope Stages and using the latter to synchronize numbering) of the Berger 1978 solution^11^. The reason for using filters instead of raw data is to minimize the possible effects of noise on the choice of start and end points to provide more objective and reproducible results, though the latter approach may also be valid. Relative timings of insolation sub-stages were determined by differencing correlative ages. Phasing diagrams show the timing differences between the forcing (insolation) and the climate response in the Leviathan and DH2-D filtered records.

## Electronic supplementary material

Supplementary Information

## References

[1] Croll, J. *Climate and Time in Their Geological Relations: A Theory of Secular Changes of the Earth’s Climate*. (D. Appleton and Co., 1875).

[2] Imbrie J (1992). On the Structure and Origin of Major Glaciation Cycles 1. Linear Responses to Milankovitch Forcing. Paleoceanography.

[3] Hays JD, Imbrie J, Shackleton NJ (1976). Variations in Earths Orbit - Pacemaker of Ice Ages. Science.

[4] Antevs E (1948). Climatic changes and pre-white man. Bulletin of the University of Utah.

[5] Benson LV (1990). Chronology of expansion and contraction of four Great Basin lake systems during the past 35,000 years. Palaeogeography, Palaeoclimatology, Palaeoecology.

[6] Broecker, W. S. & Orr, P. C. Radiocarbon Chronology of Lake Lahontan and Lake Bonneville. *Geological Society of America Bulleti*n **6**9, 1009-&, https://doi.org/10.1130/0016-7606(1958)69[1009:Rcolla]2.0.Co;2 (1958).

[7] Gilbert, G. K. 438 (Government Printing Office, Washington, 1890).

[8] Lachniet MS, Denniston RF, Asmerom Y, Polyak VJ (2014). Orbital control of western North America atmospheric circulation and climate over two glacial cycles. Nature Communications.

[9] Shakun, J. D., Burns, S. J., Clark, P. U., Cheng, H. & Edwards, R. L. Milankovitch-paced Termination II in a Nevada speleothem. *Geophysical Research Letters* **38**, 10.1029/2011GL048560 (2011).

[10] Imbrie J (1993). On the structure and origin of major glaciation cycles; 2, The 100,000-year cycle. Paleoceanography.

[11] Berger, A. Long-Term Variations of Daily Insolation and Quaternary Climatic Changes. *Journal of the Atmospheric Sciences* **35**, 2362–2367, https://doi.org/10.1175/1520-0469(1978)035<2362:ltvodi>2.0.co;2 (1978).

[12] Winograd IJ, Riggs AC, Coplen TB (1998). The relative contributions of summer and cool-season precipitation to groundwater recharge, Spring Mountains, Nevada, USA. Hydrogeology Journal.

[13] Lisiecki LE, Raymo ME (2005). A Pliocene-Pleistocene stack of 57 globally distributed benthic d^18^O records. Paleoceanography.

[14] Berger A, Loutre MF (2002). An exceptionally long interglacial ahead?. Science.

[15] Leonard EM, Plummer MA, Carrara PE (2017). Numerical modeling of the Snowmass Creek paleoglacier, Colorado, and climate in the Rocky Mountains during the Bull Lake glaciation (MIS 6). Quaternary Research.

[16] Bintanja R, van de Wal RSW, Oerlemans J (2005). Modelled atmospheric temperatures and global sea levels over the past million years. Nature.

[17] Herbert TD (2001). Collapse of the California Current during glacial maxima linked to climate change on land. Science.

[18] Marzen, R. E., DeNinno, L. H. & Cronin, T. M. Calcareous microfossil-based orbital cyclostratigraphy in the Arctic Ocean. *Quaternary Science Reviews* **149**, 109–121, http://dx.doi.org/10.1016/j.quascirev.2016.07.004 (2016).

[19] Loulergue L (2008). Orbital and millennial-scale features of atmospheric CH4 over the past 800,000 years. Nature.

[20] Luethi D (2008). High-resolution carbon dioxide concentration record 650,000-800,000 years before present. Nature (London).

[21] Wolff, E. W., Chappellaz, J., Blunier, T., Rasmussen, S. O. & Svensson, A. Millennial-scale variability during the last glacial: The ice core record. *Quaternary Science Reviews* **29**, 2828–2838, http://dx.doi.org/10.1016/j.quascirev.2009.10.013 (2010).

[22] EPICA_Group. Eight glacial cycles from an Antarctic ice core. *Nature* **429**, 623–628, http://www.nature.com/nature/journal/v429/n6992/suppinfo/nature02599_S1.html (2004). 10.1038/nature02599 15190344

[23] Oster JL, Ibarra DE, Winnick MJ, Maher K (2015). Steering of westerly storms over western North America at the Last Glacial Maximum. Nature Geoscience.

[24] Zic M, Negrini RM, Wigand PE (2002). Evidence of synchronous climate change across the northern hemisphere between the North Atlantic and the northwestern Great Basin, United State. Geology.

[25] Denniston R (2007). Synchronous millennial-scale climatic changes in the Great Basin and the North Atlantic during the last interglacial. Geology.

[26] Opel T (2017). Ground-ice stable isotopes and cryostratigraphy reflect late Quaternary palaeoclimate in the Northeast Siberian Arctic (Oyogos Yar coast, Dmitry Laptev Strait). Clim. Past.

[27] Jackson CS, Broccoli AJ (2003). Orbital forcing of Arctic climate: mechanisms of climate response and implications for continental glaciation. Climate Dynamics.

[28] Otieno FO, Bromwich DH (2009). Contribution of Atmospheric Circulation to Inception of the Laurentide Ice Sheet at 116 kyr BP. J Climate.

[29] Yin QZ, Berger A (2012). Individual contribution of insolation and CO2 to the interglacial climates of the past 800,000 years. Clim Dynam.

[30] Fisher DA, Koerner RM, Reeh N (1995). Holocene climatic records from Agassiz Ice Cap, Ellesmere Island, NWT, Canada. The Holocene.

[31] Gajewski K (2015). Quantitative reconstruction of Holocene temperatures across the Canadian Arctic and Greenland. Global and Planetary Change.

[32] MacDonald GM, Kremenetski KV, Beilman DW, Climate change (2008). and the northern Russian treeline zone. Philosophical Transactions of the Royal Society B: Biological Sciences.

[33] Funder S (2011). A 10,000-Year Record of Arctic Ocean Sea-Ice Variability—View from the Beach. Science.

[34] Briner, J. P. *et al*. Holoceneclimate change in Arctic Canada and Greenland. Q*uaternary Science Reviews* 1**47**, 340–364, http://doi.org/10.1016/j.quascirev.2016.02.010 (2016).

[35] Miller, G. H. *et al*. Temperature and precipitation history of the Arctic. *Quaternary Science Reviews* **29**, 1679–1715, http://doi.org/10.1016/j.quascirev.2010.03.001 (2010).

[36] Vinther, B. M. *et al*. Holocene thinning of the Greenland ice sheet. *Nature* **461**, 385–388, http://www.nature.com/nature/journal/v461/n7262/suppinfo/nature08355_S1.html (2009). 10.1038/nature08355 19759618

[37] Cronin, T. M. *et al*. Quaternary Sea-ice history in the Arctic Ocean based on a new Ostracode sea-ice proxy. *Quaternary Science Reviews* **29**, 3415–3429, http://doi.org/10.1016/j.quascirev.2010.05.024 (2010).

[38] Zhang Q (2010). Climate change between the mid and late Holocene in northern high latitudes - Part 2: Model-data comparisons. Clim Past.

[39] Renssen H (2005). Simulating the Holocene climate evolution at northern high latitudes using a coupled atmosphere-sea ice-ocean-vegetation model. Clim Dynam.

[40] Fischer N, Jungclaus JH (2010). Effects of orbital forcing on atmosphere and ocean heat transports in Holocene and Eemian climate simulations with a comprehensive Earth system model. Clim. Past.

[41] Sewall JO (2005). Precipitation Shifts over Western North America as a Result of Declining Arctic Sea Ice Cover: The Coupled System Response. Earth Interactions.

[42] Walland DJ, Simmonds I (1996). Modelled atmospheric response to changes in Northern Hemisphere snow cover. Climate Dynamics.

[43] Tang Q, Zhang X, Francis JA (2014). Extreme summer weather in northern mid-latitudes linked to a vanishing cryosphere. Nature Climate Change.

[44] Francis, J. A., Chan, W., Leathers, D. J., Miller, J. R. & Veron, D. E. Winter Northern Hemisphere weather patterns remember summer Arctic sea-ice extent. *Geophysical Research Letters* **36**, n/a-n/a, 10.1029/2009gl037274 (2009).

[45] Sewall JO (2005). Precipitation Shifts over Western North America as a Result of Declining Arctic Sea Ice Cover: The Coupled System Response. Earth Interactions.

[46] Köhler, P. *et al*. What caused Earth’s temperature variations during the last 800,000 years? Data-based evidence on radiative forcing and constraints on climate sensitivity. *Quaternary Science Reviews* **29**, 129–145, http://doi.org/10.1016/j.quascirev.2009.09.026 (2010).

[47] Moseley GE (2016). Reconciliation of the Devils Hole climate record with orbital forcing. Science.

[48] Laabs, B. J. C., Munroe, J. S., Best, L. C. & Caffee, M. W. Timing of the last glaciation and subsequent deglaciation in the Ruby Mountains, Great Basin, USA. *Earth and Planetary Science Letters* **361**, 16–25, http://dx.doi.org/10.1016/j.epsl.2012.11.018 (2013).

[49] Munroe JS, Laabs BJC (2013). Latest Pleistocene history of pluvial Lake Franklin, northeastern Nevada, USA. Geological Society of America Bulletin.

[50] Oviatt CG (2015). Chronology of Lake Bonneville, 30,000 to 10,000 yr BP. Quaternary Science Reviews.

[51] Vihma T (2014). Effects of Arctic Sea Ice Decline on Weather and Climate: A Review. Surveys in Geophysics.

[52] Berger M, Brandefelt J, Nilsson J (2013). The sensitivity of the Arctic sea ice to orbitally induced insolation changes: a study of the mid-Holocene Paleoclimate Modelling Intercomparison Project 2 and 3 simulations. Clim. Past.

[53] Wang T, Hamann A, Spittlehouse D, Carroll C (2016). Locally Downscaled and Spatially Customizable Climate Data for Historical and Future Periods for North America. PLoS One.

[54] Grayson, D. K. *The Great Basin: a Natural Prehistory*. (University of California Press, 2011).

[55] Waelbroeck C (2002). Sea-level and deep water temperature changes derived from benthic foraminifera isotopic records. Quaternary Science Reviews.

[56] Breitenbach SFM (2012). Constructing proxy records from age models (COPRA). Climate of the Past.

