# Supplementary material for: Arctic cryosphere and Milankovitch forcing of Great Basin paleoclimate
Source: Sci Rep. 2017 Oct 11;7:12955. doi: 10.1038/s41598-017-13279-2 (PMC5636905; doi:10.1038/s41598-017-13279-2)
Supplement: Supplementary file 1 — Supplementary Information [file 41598_2017_13279_MOESM1_ESM.doc]

# Supplemental Information

**Arctic cryosphere and Milankovitch forcing of Great Basin paleoclimate**

Matthew Lachniet^1*^, Yemane Asmerom^2^, Victor Polyak^2^, and Rhawn Denniston^3^

^1^Corresponding Author: Department of Geoscience, University of Nevada Las Vegas, 4505 Maryland Pkwy, Las Vegas, NV 89154.

^2^Department of Earth and Planetary Science, University of New Mexico, 221 Yale Blvd. NE, Albuquerque, NM 87131

^3^Department of Geology, Cornell College, 600 First Street West, Mount Vernon, Iowa 52314, USA

*Correspondence to: [matthew.lachniet@unlv.edu](mailto:matthew.lachniet@unlv.edu), 702-895-4388 (office).

## Supplemental Figures and Tables

| **Table S1. Timing and duration of events around the Last Interglacial** | | |
| --- | --- | --- |
| **Climate Events in Leviathan δ^18^O_ivc_** | **Age^1^ (yr B2k)** | **δ^18^O_ivc_** |
| ISS 6.0 | 135,100 | -13.35 |
| PGM (based on 6.0/5.5 half-height) | 163,000 to 131,100 | -12.03 |
| PGM extrema (based on 6.0/5.5 quarter-height) | 140,600 to 132,400 | -12.69 |
| ISS 5.5 | 126,400 | -10.71 |
| ISS 5.4 | 109,200 | -14.97 |
| TII (6.0/5.5 midpoint) | 131,100 | -12.03 |
| Inception (using 6.0/5.5 midpoint) | 121,300 | -12.03 |
| Inception (using 5.5/5.4 midpoint) | 120,300 | -12.84 |
| Last Interglacial Duration (*sensu stricto^2^*) | *9,800* | *yr* |
| Last Interglacial Duration (*sensu lato^3^*) | *10,800* | *yr* |
|  |  |  |
| **Climate Events in NHSI** | **Age^1^ (yr B2k)** | **W/m^2^** |
| ISS 6.0 | 139,500 | 464 |
| ISS 5.5 | 127,400 | 551 |
| ISS 5.4 | 115,600 | 440 |
| TII (6.0/5.5 midpoint) | 133,200 | 507 |
| Inception (6.0/5.5 mid-point) | 122,400 | 507 |
| Last Interglacial Duration (*sensu stricto^2^*) | *10,800* |  |
| ^1^Ages are determined on peaks and troughs of the precessional-scale 23,000 yr zero-phase band-pass filters on a 100-yr timestep. ^2^Duration based start and end δ^18^O_ivc_ and NHSI midpoints of ISS 6.0/5.5 transition; ^3^based on δ^18^O_ivc_ midpoints for start = 6.0/5.5 and end = 5.5/5.4. | | |

# Supplemental Figures

**Figure S1.** **Leviathan chronology plotted against monthly insolation at 65°N latitude.** Only summer insolation curves match the topology of the Leviathan chronology, demonstrating a strong control of Milankovitch insolation variations on Great Basin paleoclimate.


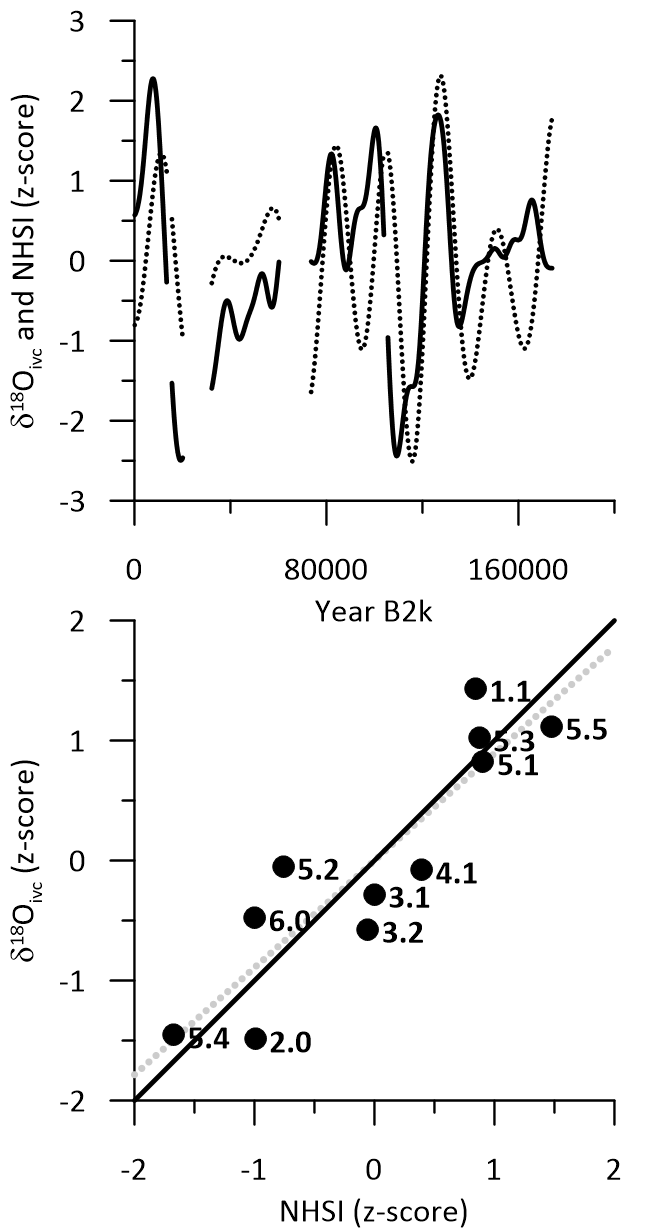


**Figure S2.** **Linear forcing between NHSI and Great Basin paleoclimate.** Top shows normalized zero-mean NHSI (dotted) and Leviathan filtered δ^18^O_ivc_ z-scores (solid). Bottom are z-scores calculated on peaks and troughs of correlation events in NHSI and filtered δ^18^O_ivc_. The slope of the best-fit line (dashed) is 0.89 and values closely plot along the linear 1:1 line (solid). The insolation curve was clipped to the growth intervals of the Leviathan chronology for comparison.


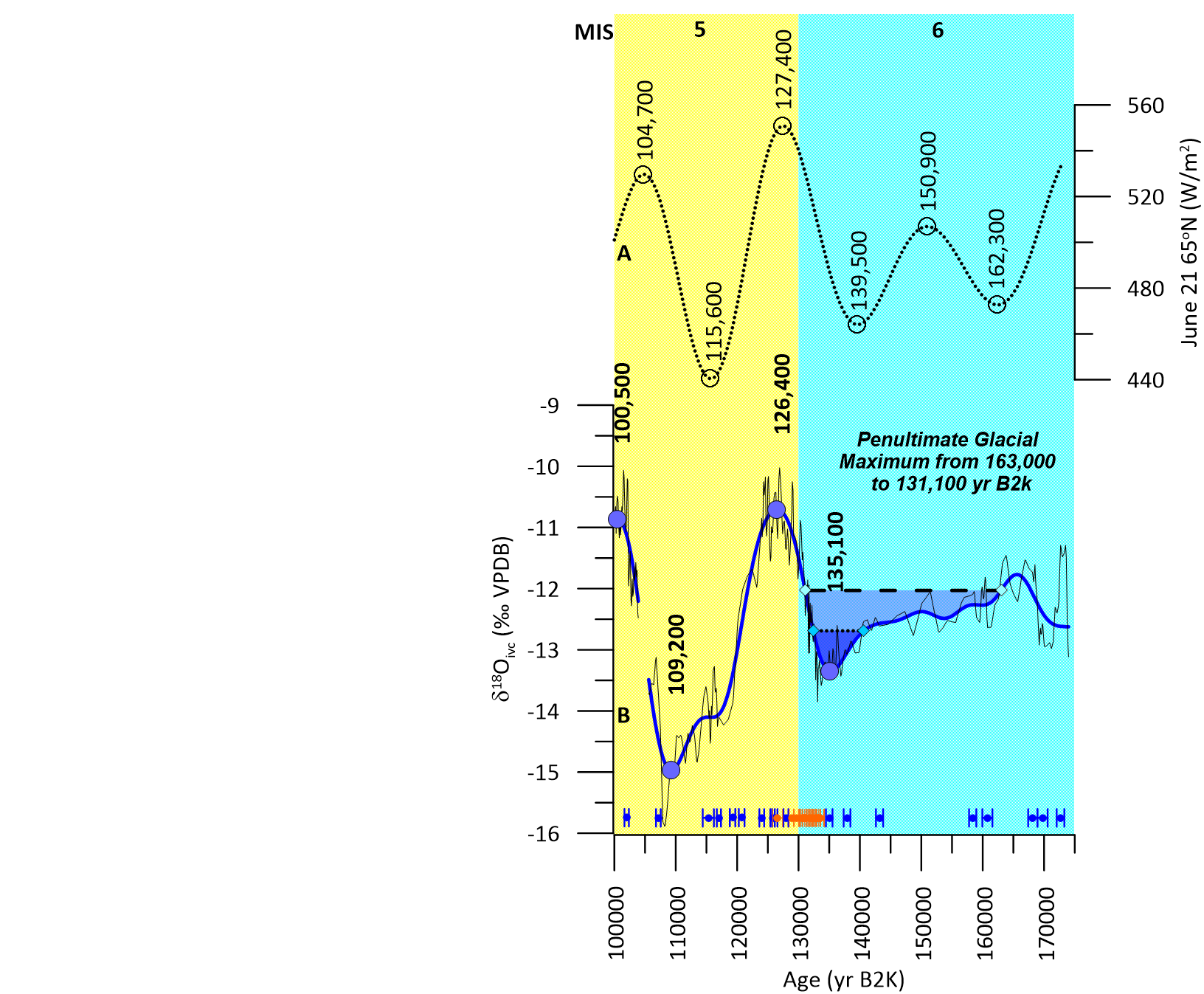


**Figure S3. Detailed time series of the Penultimate Glacial Maximum in Western North America**. The PGM lasted from 163,000 to 131,100 yr B2k and lagged NHSI forcing by ~4400 years, with the most extreme cooling centered around 135,100 yr B2k. PGM glacial records likely correlate to the timing of cold climate during the PGM. Bottom are U-series ages for Leviathan and Lehman samples LMC-21 and 14 (blue) of Ref. 8, and LC-2 (red) of Ref. 9.


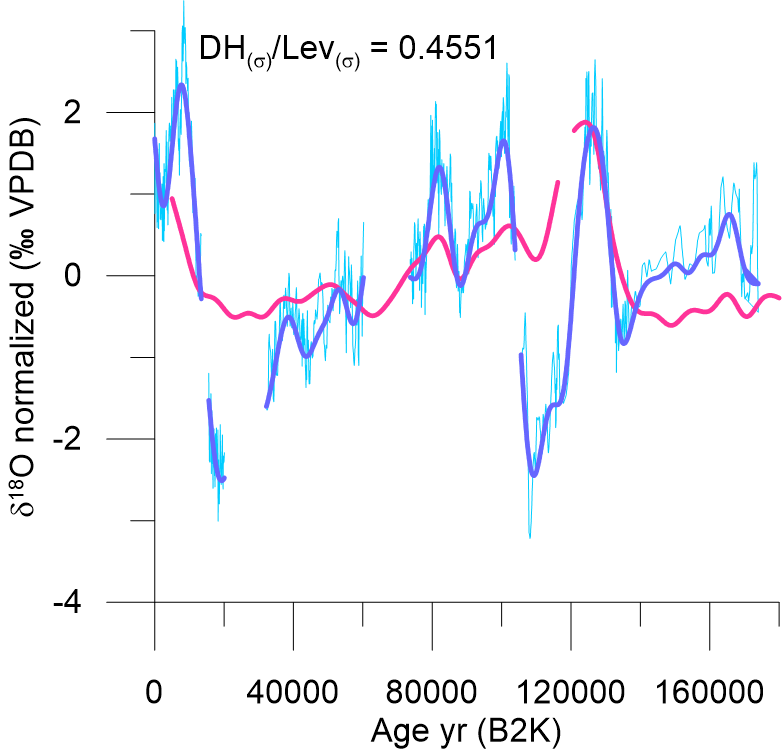


**Figure S4. Plot of Leviathan record of surface climate variations compared to the aquifer record at Devils Hole.** The reduced amplitude and lag of Devils Hole core DH2-D δ^18^O_ivc_ (pink) relative to Leviathan chronology (blue) indicates smoothing and delay of the climate signal in the aquifer feeding Devils Hole. For visual clarity, only the bandpass filtered δ^18^O_ivc_ for DH2-D is shown. The ratio of standard deviations (σ) for the two records is 0.4551.


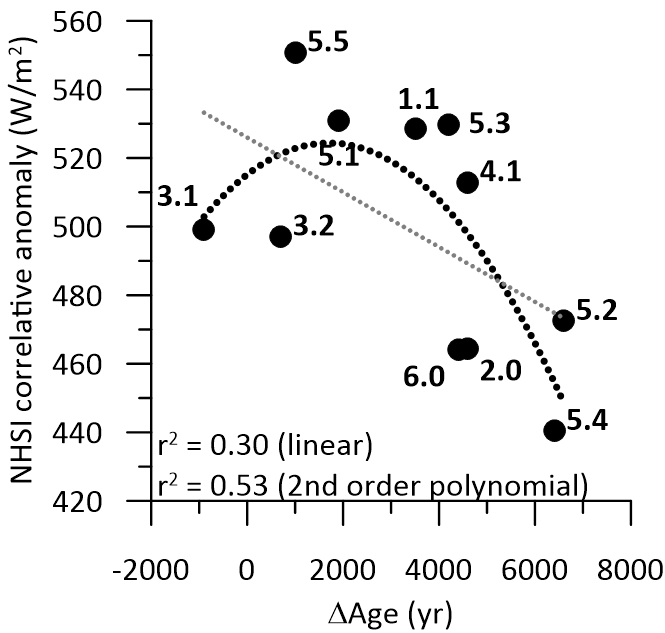


**Figure S5. Relationship between lags and NHSI.** The lags of Leviathan δ^18^O_ivc_ behind ages of correlative NHSI peaks (see Table 1) are largest during insolation minima during the last and penultimate glacial maximums, and NHSI minima during Marine Isotope Stage 5. Labels are insolation sub-stages.
